# Surgical versus nonsurgical treatment for scaphoid waist fracture with slight or no displacement: A meta-analysis and systematic review

**DOI:** 10.1097/MD.0000000000013266

**Published:** 2018-11-30

**Authors:** Hangyu Li, Wenlai Guo, Shanshan Guo, Shishun Zhao, Rui Li

**Affiliations:** a Center for Applied Statistical Research and College of Mathematics, Jilin University; b Hand & Foot Surgery and Reparative & Reconstruction Surgery Center, The Second, Hospital of Jilin University, Changchun, China.

**Keywords:** meta-analysis, scaphoid waist fracture, surgical versus nonsurgical

## Abstract

**Background:**

Surgical or nonsurgical treatment for scaphoid waist fracture with slight or no displacement is still controversial. This study compared the efficacy of the 2 methods through meta-analysis to provide a reference for the choice of clinical treatment options.

**Methods:**

Two individuals independently searched for relevant RCTs and cohort studies from PubMed (1946–February 2018), Embase (1946–February 2018), and Cochrane library (1997–February 2018). After quality assessment and data extraction, Stata 14 software was used for combining the effect size, testing heterogeneity, and studying bias. GRADEpro was used to rate the level of evidence.

**Results:**

Ten RCTs and 4 cohort studies with 765 patients were included. No statistical difference in satisfaction, pain, and Disability of the Arm, Shoulder, and Hand score was found after surgical and nonsurgical treatments. Compared with nonsurgical treatment, surgical treatment shortened the time to union (SMD = −5.01, 95% CI: −7.47 to −2.58, *P = *.000), decreased the convalescence (SMD = −2.09, 95% CI: −3.08 to −1.11, *P = *.000), and reduced the incidence of nonunion (RR = 0.47, 95% CI: 0.24–0.90), *P = *.023). Subgroup analyses showed that the percutaneous fixation treatment can shorten the time to union [SMD = −1.82, 95%CI (−2.22 to −1.42), *P = *= .000] and the convalescence (SMD = −4.26, 95%CI: −6.16 to −2.35, *P = *= .054), and open reduction fixation treatment can reduce the incidence of nonunion (RR = 0.20, 95%CI: 0.06–0.69, *P = *= .01).

**Conclusion:**

For scaphoid waist fractures with slight or no displacement, there was no statistical difference in patient satisfaction, pain, and The Disability of the Arm, Shoulder, and Hand scores between surgical treatment and nonsurgical treatment. Closed surgical treatment can shorten the time to union and convalescence, and open reduction can reduce the incidence of nonunion. On the basis of this conclusion, chief physicians can consider which treatment to use according to the patient's clinical situation and their subjective intention.

## Abstract 2

Supplemental Digital Content is available in the text

## 1 Introduction

Scaphoid waist fracture is a common type of wrist fracture,^[1]^ accounting for 51% to 90% of wrist fractures and 2% to 7% of total body fractures.^[2,3]^ Because of the particular distribution of scaphoid blood vessels, blood circulation is often blocked after fracture of the scaphoid.^[4,5]^ If blood circulation cannot be properly restored, severe wrist dysfunction can develop.^[6]^

The best treatment for scaphoid waist fracture with slight or no displacement has been the focus of controversy.^[7,8]^ Some early scholars have suggested that nonsurgical treatment, such as long-arm or short-arm plaster support and brace fixation for 9 to 12 weeks^[9–11]^ until fracture union, which has a union rate of 90% to 95%.^[11–14]^ Above all, nonsurgical treatment is non-invasive and has a low economic burden. In recent years, some scholars have advocated the use of surgical treatment,^[15,16]^ for which the union rate is almost 100%.^[4,6,17,18]^ Surgical treatment is characterized by performing rehabilitation postoperatively, avoiding joint stiffness caused by long-term plaster fixation,^[6,19]^ and reducing the incidence of nonunion and malunion of the scaphoid bone.^[16,20]^

There was no significant difference in the union rate between the 2 treatment methods. However, the differences in the trauma caused by treatment, degree of satisfaction caused by postoperative joint stiffness, and pain were significant. This is an important factor affecting patients’ quality of life and the main purpose of our treatment. Considerably insufficient attention to postoperative satisfaction and pain cannot provide reliable clinical evidence in studies regarding scaphoid waist fracture with slight or displacement.

At present, only 3 meta-analyses^[21–23]^ have reported on this topic, but because of the lack of basic literature and errors in document inclusion and data extraction,^[21]^ the validity of the evidence is insufficient. Thus, we searched for recent updates that included randomized, controlled trials (RCTs) and cohort studies to increase the number of primary documents.^[24–27]^ We performed a meta-analysis of outcome measures, such as patient satisfaction, pain, The Disability of the Arm, Shoulder, and Hand (DASH) scores, and fracture union in patients with scaphoid waist fracture with slight or no displacement between surgical and nonsurgical treatments. In addition, we performed a subgroup analysis on time to union, the convalescence, and incidence of nonunion between the surgical treatment groups (open reduction surgery versus percutaneous fixation surgery).

## 2 Methods

### 2.1 Search strategy

We performed this meta-analysis and systematic review following the guidelines of the Preferred Reporting Items for Systematic Reviews and Meta-analyses statement,^[28]^ and we retrieved articles from PubMed (1946–February 2018), Embase (1946–February 2018), and Cochrane library (1997–February 2018). The keywords used were “scaphoid bone;” “fractures, bone;” and “surgical procedures, operative” and synonyms for these terms. The references for relevant reviews and systematic reviews were manually retrieved. All analyses were based on previous published studies; thus, no ethical approval and patient consent are required.

### 2.2 Basic PubMed search

((“Surgical Procedures, Operative”[Mesh] OR ((((((((((Operative Surgical Procedure[Title/Abstract] OR Operative Surgical Procedures[Title/Abstract]) OR ((“methods”[Subheading] OR “methods”[All Fields] OR “procedures”[All Fields] OR “methods”[MeSH Terms] OR “procedures”[All Fields]) AND Operative Surgical[Title/Abstract])) OR ((“surgical procedures, operative”[MeSH Terms] OR (“surgical”[All Fields] AND “procedures”[All Fields] AND “operative”[All Fields]) OR “operative surgical procedures”[All Fields] OR (“surgical”[All Fields] AND “procedure”[All Fields]) OR “surgical procedure”[All Fields]) AND Operative[Title/Abstract])) OR Operative Procedures[Title/Abstract]) OR Operative Procedure[Title/Abstract]) OR ((“methods”[MeSH Terms] OR “methods”[All Fields] OR “procedure”[All Fields]) AND Operative[Title/Abstract])) OR ((“methods”[Subheading] OR “methods”[All Fields] OR “procedures”[All Fields] OR “methods”[MeSH Terms] OR “procedures”[All Fields]) AND Operative[Title/Abstract])) OR ((“methods”[MeSH Terms] OR “methods”[All Fields] OR “procedure”[All Fields]) AND Operative Surgical[Title/Abstract])) OR (Surgery,[All Fields] AND Ghost[Title/Abstract])) OR Ghost Surgery[Title/Abstract])) AND (“Fractures, Bone”[Mesh] OR ((((((((((((((Broken Bones[Title/Abstract] OR ((“bone and bones”[MeSH Terms] OR (“bone”[All Fields] AND “bones”[All Fields]) OR “bone and bones”[All Fields] OR “bone”[All Fields]) AND Broken[Title/Abstract])) OR ((“bone and bones”[MeSH Terms] OR (“bone”[All Fields] AND “bones”[All Fields]) OR “bone and bones”[All Fields] OR “bones”[All Fields]) AND Broken[Title/Abstract])) OR Broken Bone[Title/Abstract]) OR Bone Fractures[Title/Abstract]) OR Bone Fracture[Title/Abstract]) OR Fracture, Bone[Title/Abstract]) OR Spiral Fractures[Title/Abstract]) OR (Fracture,[All Fields] AND Spiral[Title/Abstract])) OR (Fractures,[All Fields] AND Spiral[Title/Abstract])) OR Spiral Fracture[Title/Abstract]) OR Torsion Fractures[Title/Abstract]) OR (Fracture,[All Fields] AND Torsion[Title/Abstract])) OR (Fractures,[All Fields] AND Torsion[Title/Abstract])) OR Torsion Fracture[Title/Abstract]))) AND (“Scaphoid Bone”[Mesh] OR (((((((((“bone and bones”[MeSH Terms] OR (“bone”[All Fields] AND “bones”[All Fields]) OR “bone and bones”[All Fields] OR “bone”[All Fields]) AND Scaphoid[Title/Abstract]) OR ((“bone and bones”[MeSH Terms] OR (“bone”[All Fields] AND “bones”[All Fields]) OR “bone and bones”[All Fields] OR “bones”[All Fields]) AND Scaphoid[Title/Abstract])) OR Scaphoid Bones[Title/Abstract]) OR Os Naviculare Manus[Title/Abstract]) OR Os Scaphoideum[Title/Abstract]) OR Navicular Bone of Hand[Title/Abstract]) OR ((“hand”[MeSH Terms] OR “hand”[All Fields]) AND Navicular Bone[Title/Abstract])) OR ((“hand”[MeSH Terms] OR “hand”[All Fields]) AND Navicular Bones[Title/Abstract])))

### 2.3 Eligibility criteria

#### 2.3.1 Inclusion criteria

(1) *Design type*: RCT and cohort studies concerning surgical or nonsurgical treatment for scaphoid fracture in English and Chinese were included.
(2) *Participants*: Patients with no displacement or < 1 mm of displacement, a scaphoid waist fracture, and follow-up for at least 2 months were included.
(3) *Interventions*: Patients in the surgical treatment group underwent open reduction and percutaneous internal fixation; whereas, those in the nonsurgical treatment group received different types and lengths of plaster or braces to fix the fracture.

#### 2.3.2 Exclusion criteria

The exclusion criteria were as follows:

(1) scaphoid tubercle fracture, proximal fracture, comminuted fracture, open scaphoid fracture, and scaphoid waist fracture with displacement >1 mm;
(2) combination of ligament injuries and other wrist fractures;
(3) fractures for >2 weeks;
(4) previous wrist injury or surgical history, signs of osteoarthritis on the x-ray of the wrist, and previous disease that affects fracture union; and
(5) case reports, cadaver research, and biomechanical research.

Two investigators (HL and WG) independently extracted data for cross-checking; if there was disagreement, the third investigator (SZ) would make a conclusion after discussion between the 2 parties.^[29]^

### 2.4 Assessment of methodological quality

Two investigators (HL and WG) independently assessed the quality of the included literature: RCTs were assessed by the modified Jadad scale, with scores <4 indicating low quality.^[30]^ Cohort studies were assessed using the Newcastle–Ottawa scale (NOS), with scores <5 indicating low quality. After disagreement was discussed by the 2 parties, the third investigator (RL) made the final decision.

### 2.5 Outcome measures

Indicators for assessing the efficacy included the following.

(1) *Degree of patient satisfaction*: Patient's ultimate satisfaction with treatment outcomes was based on a self-assessment.
(2) *Feeling of pain*: Using the visual analog scale (VAS) score, the degree of pain was represented by a number from 0 to 10, with 0 indicating no pain and 10 indicating the most pain.^[31]^
(3) *Functional evaluation*: The DASH score was used for the functional evaluation. It is a patient survey questionnaire containing 30 items on features and symptoms of upper limb disease, with scores ranging from 0 to 100; high scores represent poor function.^[32]^
(4) *Time to union*: Good trabecular bone structure at the transverse site revealed on images was considered fracture union.^[33]^
(5) *Convalescence*: The times until the patient returned to work and activity were assessed (e.g., athletes’ return to previous training activities).
(6) *Incidence of nonunion*: Nonunion was defined as signs of non-union at the fracture site more than one-half year postoperatively and during the 3-month follow-up (e.g., the fracture was still not healed).

### 2.6 Data collection

Two investigators (HL and WG) independently extracted data that contained the first author, year of publication, sample size, sex, intervention measures, follow-up time, patients’ satisfaction, patients’ pain, DASH scores, time to union, convalescence, incidence rate of nonunion.

We sent emails to the author of the relevant study in an attempt to obtain unpublished raw data, but no responses were received. When the standard deviation (SD) was not provided, range and median were used to estimate it,^[34]^ or the SD was estimated from the confidence interval (CI) using the method described in the Cochrane Handbook for Systematic Reviews of Interventions.^[29]^

### 2.7 Statistical analysis

All data were analyzed using Stata 14 software (Stata Corp.). The heterogeneity among the included studies was analyzed using the chi-square test. The random effect model was used when *I*^2^ >50%; otherwise, the fixed effect model was used for merging analysis.^[35]^ Dichotomous variables are represented by the relative risk (RR). Continuous therapeutic effect variables are expressed by standardized mean difference. The 95% CI estimates and hypothesis test results for each variable are listed in the forest map.

Heterogeneity was obvious when the *P*-value was <.05 (chi-square test) and *I*^2^ was >50%.^[36]^ Sources of heterogeneity included diagnostic methods, reported results, and differences in surgical techniques. Because of the study design, these variables could not be controlled.^[37]^ When *I*^2^ >50%, the included studies were removed one by one to the sensitivity analysis that was conducted to determine the sources of heterogeneity. For result indicators of no less than 8 primary documents, subgroup analysis was performed according to different methods of percutaneous fixation and open reduction and internal fixation. For outcome measures of >10 primary documents, the publication bias test was performed using a funnel plot and Egger's test. Finally, GRADEpro software (Grade Working Group) was used to verify the quality of evidence by classifying the results in order to provide reliable evidence for clinical selection.

## 3 Results

### 3.1 Search results and characteristics of the selected studies

**Figure 1:**
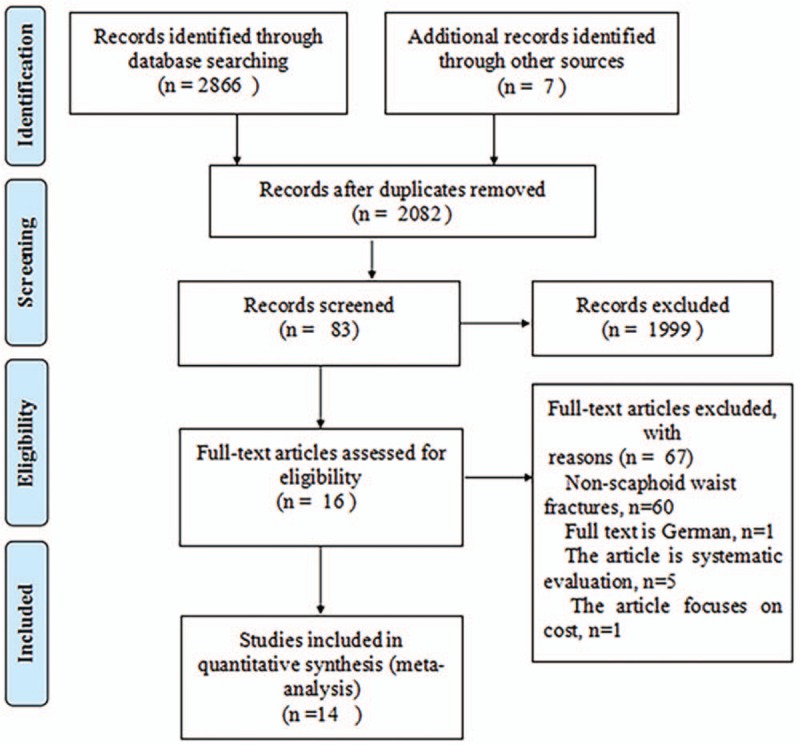
Flowchart of selection of studies.

Of the 2873 potentially suitable studies, 14 met our criteria (Fig. 1),^[24–27,38–47]^ including 10 RCTs^[25,27,39–42,44–47]^ and 4 cohort studies.^[24,26,38,43]^ Among 765 patients, 384 were treated with surgery, and 381 patients underwent nonsurgical treatment. Surgical treatment comprised open reduction and internal fixation (n = 195) and percutaneous fixation (n = 189). Nonsurgical treatment included short-arm plaster fixation (n = 174), long-arm plaster fixation with the thumb (n = 14) and short-arm plaster fixation with the thumb (n = 103). The follow-up period for the basic studies ranged from 5 weeks to 13 years. The basic characteristics of the included studies are shown in Table 1.

**Table 1:**
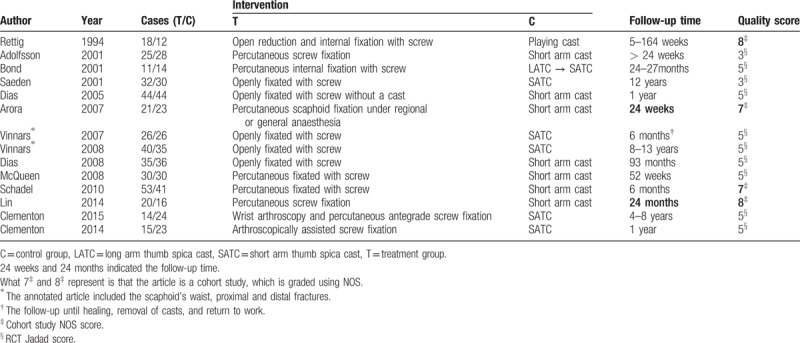
Main characteristics of all eligible studies included in the analysis.

The quality of each RCT was assessed according to the Jadad scale. Eight studies^[25,27,40,42,44–47]^ had high quality, all of which used allocation concealment with sealed envelopes. Two studies^[39,41]^ had low quality. There was no double-blind study. According to the NOS scale, the quality of each cohort study was scored, and 4 studies^[24,26,38,43]^ had high quality.

Two studies^[42,46]^ used the same participants but recorded different outcome indicators so all the patients from these studies were included in the present study. In 2008, Vinnars et al^[45]^ expanded the sample size based on their previous study,^[44]^ and increased and changed part of the outcome indicators; thus, those 2 studies were included. The same outcome measures were based on the later report.^[45]^ Two other studies^[25,27]^ had the same issue and were included at the same time.

### 3.2 Comparison of operative and nonoperative treatment on patients’ satisfaction

**Figure 2:**
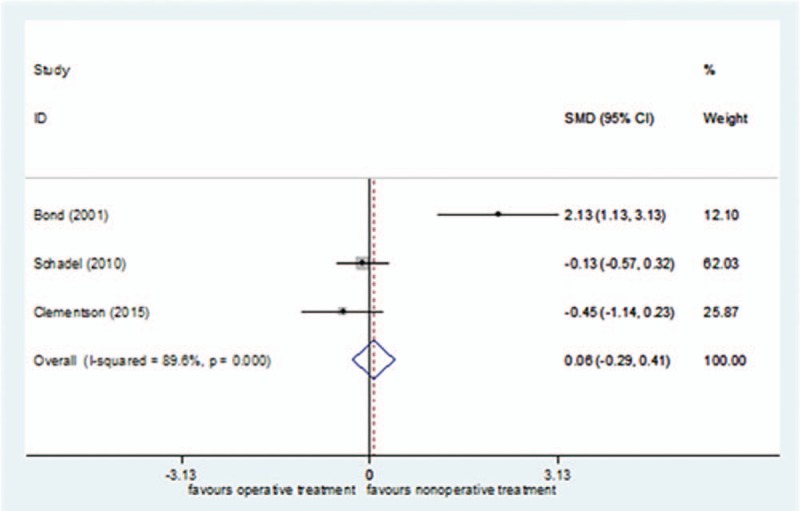
Forest plot for patients’ satisfaction.

Three studies^[24,27,40]^ reported patients’ satisfaction, including 67 cases in the surgical treatment group and 72 cases in the nonsurgical treatment group, for a total of 139. *I*^2^ = 89.6%, so the random effect model was selected. There was no significant difference in the patients’ satisfaction between the surgical treatment group and the nonsurgical treatment group[standard mean difference (SMD) = 0.06, 95%CI (−0.29–0.41)] (Fig. 2). In the sensitivity analysis, Bond et al's study^[40]^ was excluded, and *I*^2^ was reduced to 0% (*P = *.433). Using the fixed effect model, the conclusion was unchanged [SMD = 0.22, 95%CI (−0.59–0.15), *P = *= .240].

The GRADEpro system's quality classification of Patients’ satisfaction was low (S1 Appendix).

### 3.3 Comparison of operative and non-operative treatment on patients’ pain

**Figure 3:**
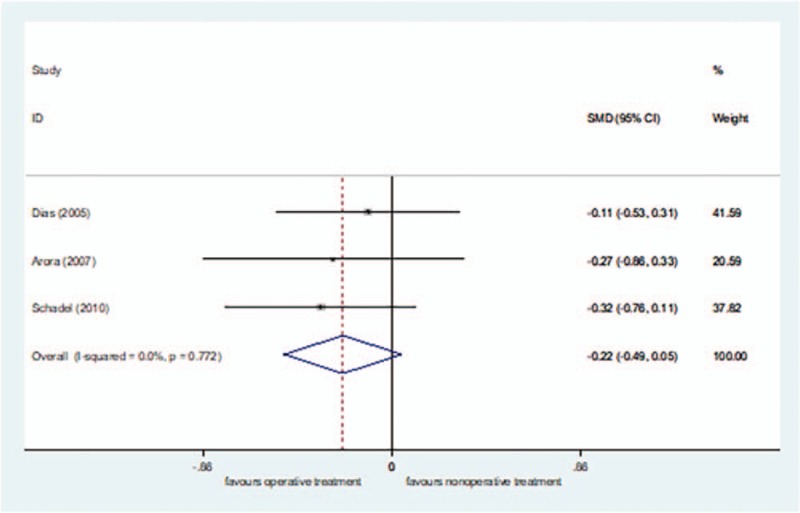
Forest plot for patients’ pain at short-term follow-up.

Three studies^[24,42,43]^ reported patients’ pain, including 113 cases in the surgical treatment group and 102 cases in the nonsurgical treatment group, for a total of 215. *I*^2^ = 0% (*P = *= .772) of VAS score comparison in short-term follow-up, so the fixed effect model was selected. There was no significant difference in the patients’ pain in short-term follow-up between the surgical treatment group and the nonsurgical treatment group [SMD = − 0.22, 95%CI (−0.49–0.05), *P = *= .107] (Fig. 3).

**Figure 4:**
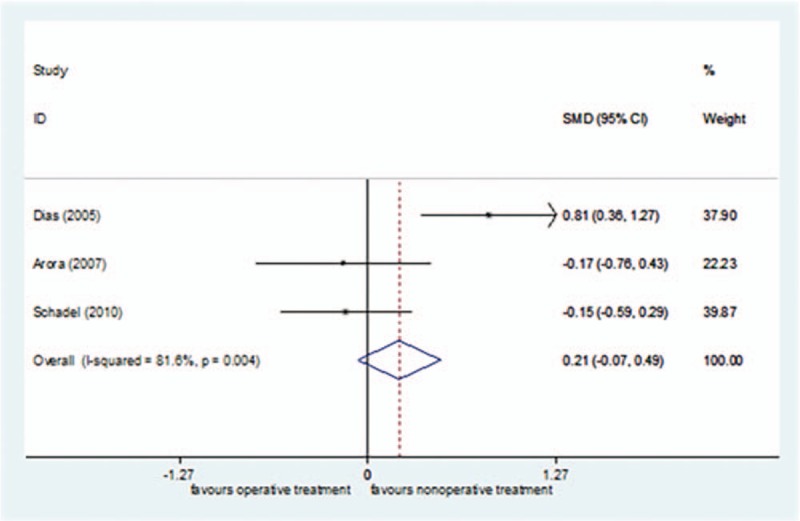
Forest plot for patients’ pain at end-follow-up.

*I*^2^ = 81.6% of VAS score comparison in long-term follow-up, so the random effect model was selected. There was no significant difference in the patients’ satisfaction in long-term follow-up between the surgical treatment group and the nonsurgical treatment group [SMD = 0.21, 95%CI (−0.07–0.49)] (Fig. 4). In the sensitivity analysis, Dias et al.'s study^[42]^ was excluded, and *I*^2^ was reduced to 0% (*P = *.963). Using the fixed effect model, the conclusion was unchanged [SMD = −0.16, 95%CI (−0.51–0.20), *P = *= .390].

The GRADEpro system's quality classification of Patients’ Pain was low (S2 Appendix).

### 3.4 Comparison of operative and nonoperative treatment on DASH scores

**Figure 5:**
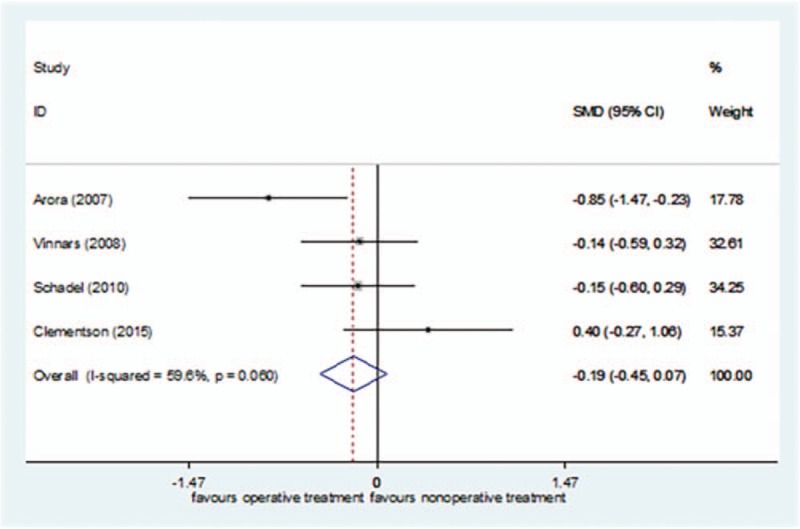
Forest plot for DASH scores. DASH = The Disability of the Arm, Shoulder, and Hand.

Four studies^[24,27,43,45]^ reported DASH scores, including 102 cases in the surgical treatment group and 94 cases in the nonsurgical treatment group, for a total of 196. *I*^2^ = 59.6%, so the random effect model was selected. There was no significant difference in the DASH scores between the surgical treatment group and the nonsurgical treatment group [SMD = −0.19, 95%CI (−0.45–0.07)] (Fig. 5). In the sensitivity analysis, Arora et al's study^[43]^ was excluded, and *I*^2^ was reduced to 3.5% (*P = *.433). Using the fixed effect model, the conclusion was unchanged [SMD = −0.04, 95%CI (−0.33–0.24), *P = *= .769].

The GRADEpro system's quality classification of DASH Scores was low (S3 Appendix).

### 3.5 Comparison of operative and nonoperative treatment on time to union

**Figure 6:**
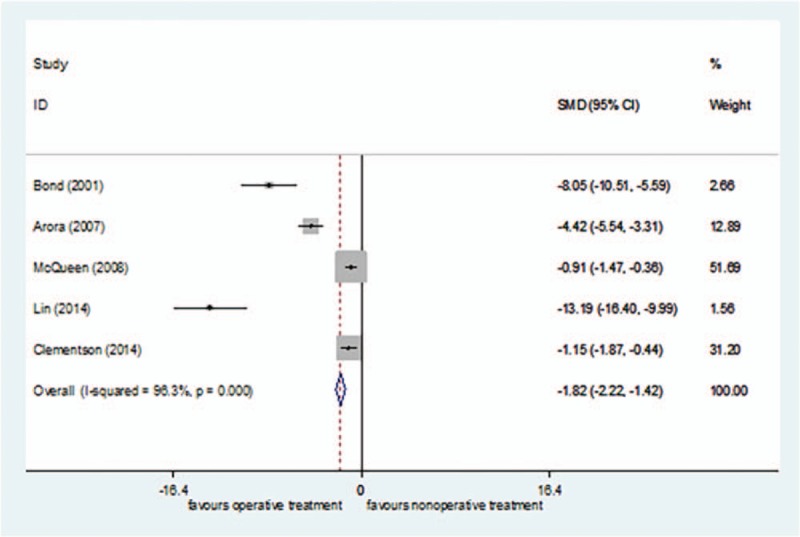
Forest plot for time to union of random.

Five studies^[25,26,40,43,47]^ reported time to union, including 95 cases in the surgical treatment group and 102 cases in the nonsurgical treatment group, for a total of 197. *I*^2^ = 96.3% (*P = *< 0.00001), so the random effect model was selected. The time to union was shorter in the surgical treatment group than in the nonsurgical treatment group [SMD = −1.82, 95%CI (−2.22 to −1.42), *P = *= .000] (Fig. 6). The sensitivity analysis did not find any sources of heterogeneity.

The GRADEpro system's quality classification of Time to Union was moderate (S4 Appendix).

### 3.6 Comparison of operative and non-operative treatment on convalescence

**Figure 7:**
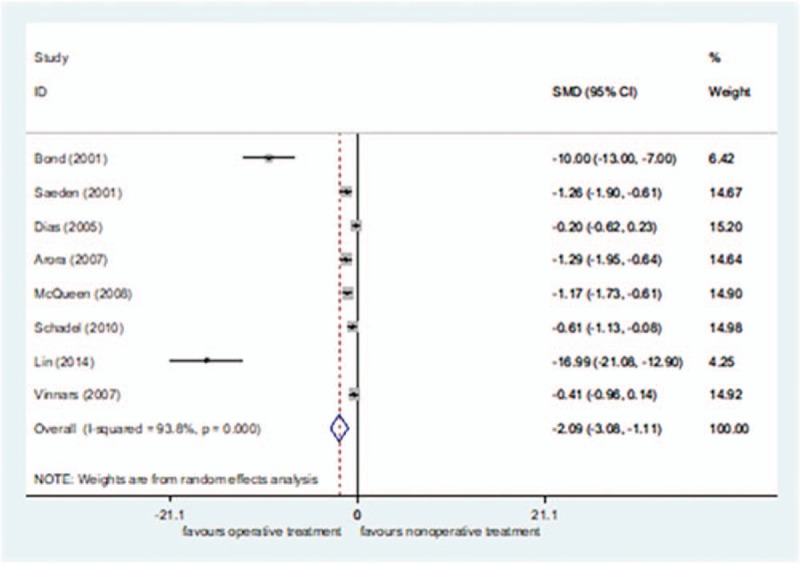
Forest plot for convalescence of random.

Eight studies^[24,26,40–44,47]^ reported convalescence, including 208 cases in the surgical treatment group and 196 cases in the nonsurgical treatment group, for a total of 404. *I*^2^ = 93.8% (*P = *< 0.00001), so the random effect model was selected. The convalescence was shorter in the surgical treatment group than in the nonsurgical treatment group[SMD = −2.09, 95%CI (−3.08 to −1.11), *P = *= .000] (Fig. 7). The sensitivity analysis did not find any sources of heterogeneity.

**Figure 8:**
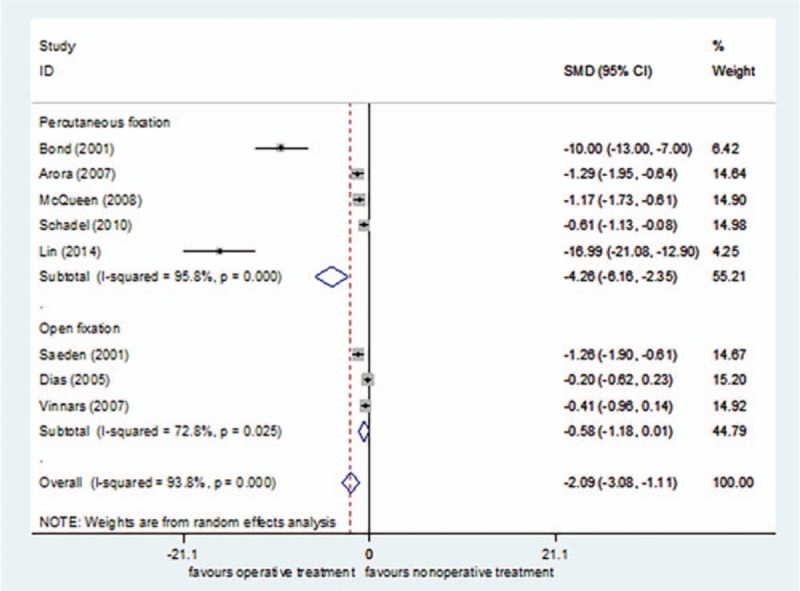
Subgroup analysis for convalescence.

The subgroup analysis of surgical methods demonstrated that the convalescence was shorter in the percutaneous fixation group than in the nonsurgical treatment group [SMD = −4.26, 95%CI (−6.16 to −2.35), *P = *= .054]. There was no significant difference in the convalescence between the open reduction fixation group and nonsurgical treatment group [SMD = −0.58, 95%CI (−1.18–0.01), *P = *= .000] (Fig. 8).

The GRADEpro system's quality classification of Convalescence was moderate (S5 Appendix).

### 3.7 Comparison of operative and nonoperative treatment on the incidence rate of nonunion

**Figure 9:**
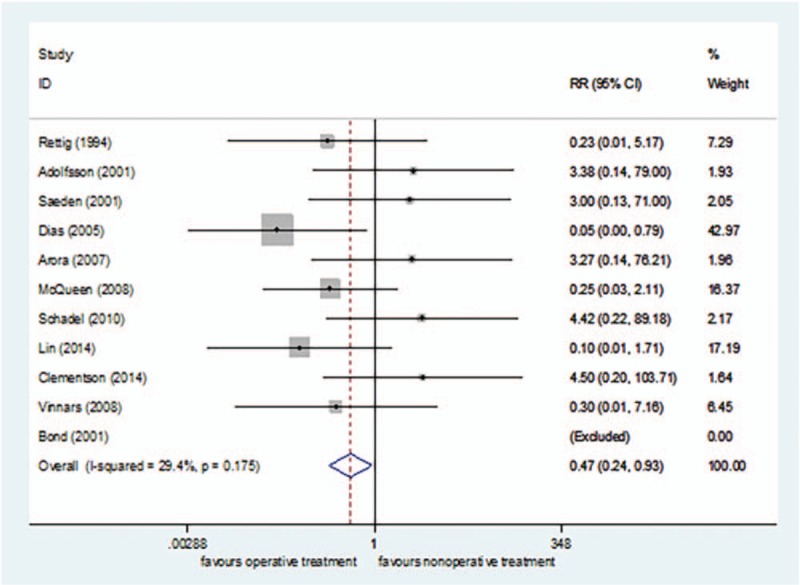
Forest plot for the incidence rate of nonunion.

Eleven studies^[24–26,38–43,45,47]^ reported the incidence rate of nonunion, including 282 cases in the surgical treatment group and 282 cases in the nonsurgical treatment group, for a total of 564. *I*^2^ = 29.4% (*P = *= .175), so the fixed effect model was selected. The incidence rate of nonunion was lower in the surgical treatment group than in the nonsurgical treatment group[RR = 0.47, 95%CI (0.24–0.93), *P = *= .03] (Fig. 9).

**Figure 10:**
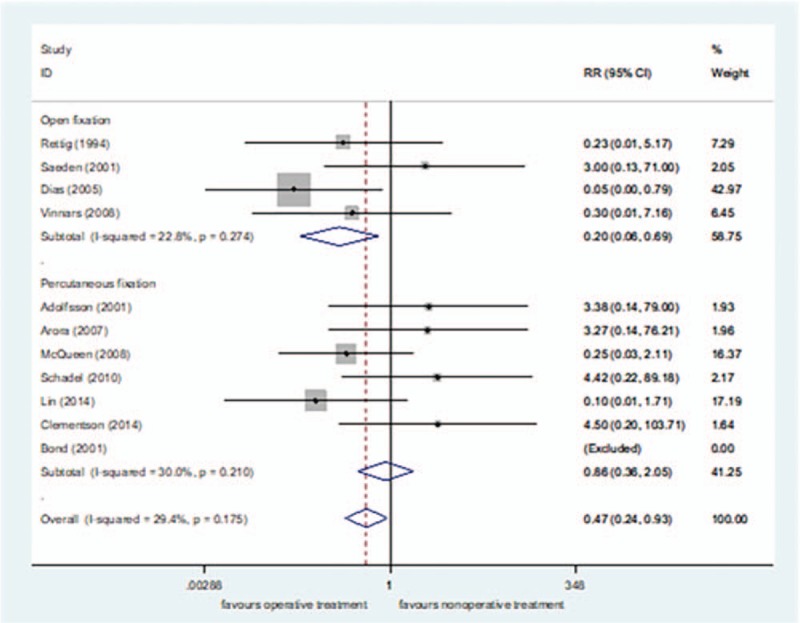
Subgroup analysis for the incidence rate of nonunion.

**Figure 11:**
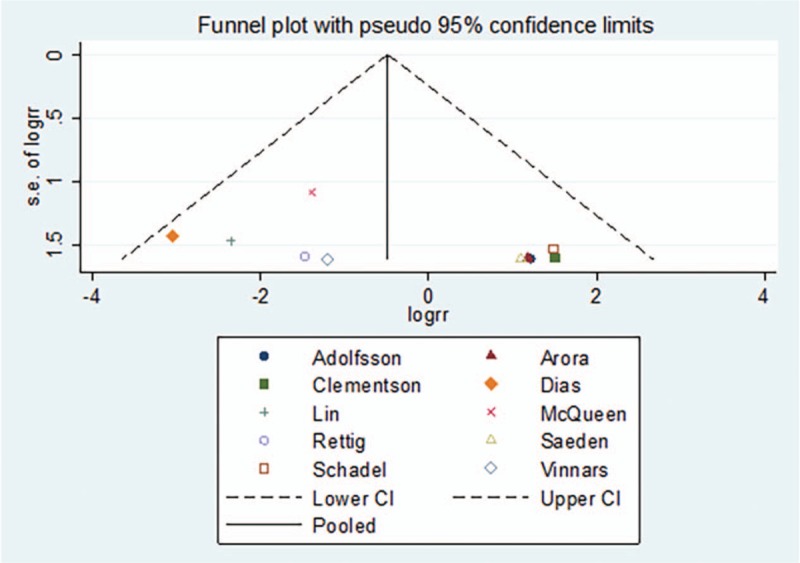
Funnel plot for the incidence rate of nonunion.

**Figure 12:**
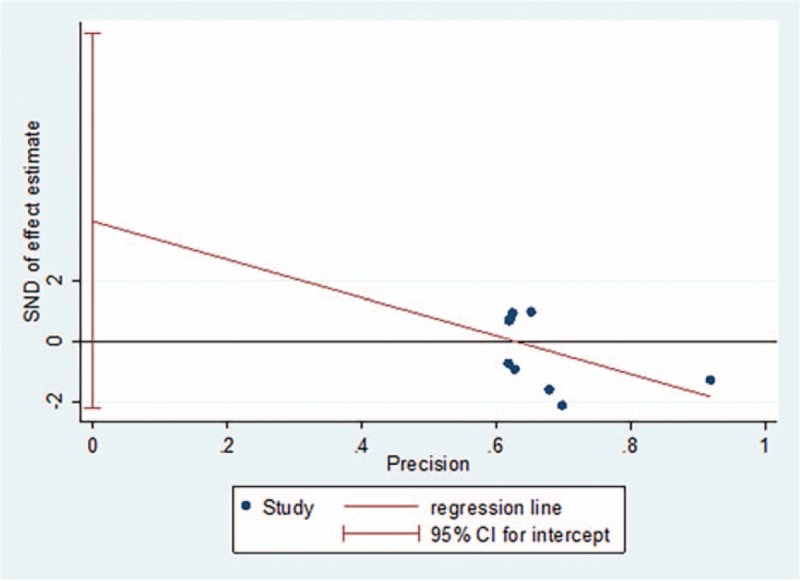
Egger's regression plot for the incidence rate of nonunion.

The subgroup analysis of surgical methods demonstrated that the incidence of nonunion was lower in the open reduction fixation group than in the nonsurgical treatment group[RR = 0.20, 95%CI (0.06–0.69), *P = *= .01] (Fig. 10). There was no significant difference in the incidence of nonunion between the percutaneous fixation group and nonsurgical treatment group[RR = 0.86, 95%CI (0.36–2.05), *P = *= .74] (Fig. 10). The funnel plot was symmetric, with an Egger's test result of *P = *.179 (95% CI [−2.24–10.15]), with no apparent publication bias (Figs. 11 and 12).

The GRADEpro system's quality classification of the Incidence Rate of Nonunion was moderate (S6 Appendix).

## 4 Discussion

### 4.1 Key findings

Our results showed that the degree of patient satisfaction, pain, and DASH scores were not significantly different between surgical and nonsurgical treatments for scaphoid waist fracture with slight or no displacement. The time to union was faster, recovery time was shorter, and incidence of nonunion was lower in the surgical treatment group than in the nonsurgical treatment group. That is, surgical treatment was more advantageous in the treatment of scaphoid waist fracture with slight or no displacement. The GRADEpro system^[48,49]^ rated the quality of the DASH score, time to union, convalescence, and incidence of nonunion as moderate evidence, and patient satisfaction and pain as low.

### 4.2 Sensitivity analysis

Previous meta-analysis and systematic reviews have lacked effective research on patient satisfaction. Suh et al^[22]^ and Symes et al^[23]^ only conducted a qualitative systematic evaluation and lacked a quantitative meta-analysis. Although Buijze et al^[21]^ tried to perform quantitative analysis, they only included Bond et al's study.^[40]^ Moreover, the five-point satisfaction rating (score, 0–4) was incorrectly described as a six-point system (score, 0–5). This study found that the overall satisfaction of surgical treatment and nonsurgical treatment was high, and there was no statistical difference. The participants in Bond et al's study^[40]^ had different job functions. They were young military personnel aged 18–34 years. Their postoperative recovery was quick so their satisfaction was generally high. This may be the main source of heterogeneity.

Surgical treatment and nonsurgical treatment showed no significant difference in pain at the short-term and final follow-ups. Dias et al^[42]^ used an alternative approach called “aggressive conservative treatment” to achieve effective therapeutic effects, and this may be also a source of heterogeneity. A treatment protocol^[50]^ also proposed “non-invasive surgery,” in which all non-displaced scaphoid fractures were first treated with nonsurgical treatment, and if there was no sign of union at the fracture site at week 12, further surgery was considered.

The time to union and convalescence was shorter after surgical treatment than after nonsurgical treatment. The main reason for this finding may be as follows: long-term plaster fixation leads to joint stiffness, muscle weakness, and failure to effectively pressurize the fracture site.^[51–53]^ In the study of time to union, the surgical treatment group was treated with percutaneous fixation without open reduction and internal fixation. Thus, the time to union of percutaneous fixation was shorter than that of nonsurgical treatment. The clinical heterogeneity of this outcome measure was large, mainly because the time to union, as an important indicator reflecting the therapeutic effect, was difficult to measure, and imaging instruments with different precisions resulted in inaccurate results.^[37,54]^ For example, Bond et al^[40]^ used x-rays to evaluate union once every 2 weeks. Lin et al^[26]^ used computed tomography scans and Mimics software to assess union. With regard to the convalescence, subgroup analysis according to the surgical method found that the convalescence was shorter in the percutaneous fixation group than in the nonsurgical treatment group, and there was no statistical difference in the convalescence between the open reduction fixation group and nonsurgical treatment group. The possible explanations for this finding are that percutaneous fixation of scaphoid fractures provides effective pressure on the fracture ends, does not require the articular capsule to be cut, causes less damage to regional soft tissue, and does not damage the peripheral ligaments and nerves, thereby avoiding further aggravation of the wrist injury and obviously shortening the convalescence.^[23,55,56]^ Open reduction and internal fixation may increase injury to the associated ligaments and nerves, which is not conducive to the healing of soft tissue. Postoperatively, it is often necessary to supplement with external fixation, which results in joint stiffness and makes surgery less effective in shortening the convalescence.^[57–59]^

The incidence of nonunion was lower after surgical treatment (2.36% [7/296]) than after nonsurgical treatment (6.55% [19/290]). Vinnars et al's study^[45]^ included the scaphoid waist and distal and proximal ends. It was not possible to extract only part of the scaphoid wrist data. Therefore, after retaining the data analysis, the data were carefully eliminated for sensitivity analysis, and it was found that the conclusion was unchanged. The subgroup analysis found that the incidence of nonunion was lower in the open reduction fixation group than in the nonsurgical treatment group; there was no significant difference in the incidence of nonunion between the percutaneous fixation group and nonsurgical treatment group. Previously, we empirically and mistakenly considered percutaneous fixation as a minimally invasive surgery that can achieve the goal of pressurizing the fracture end without destroying blood flow of the soft tissue around the fracture site, and promote fracture union. Alshryda et al^[50]^ also unexpectedly discovered that contrary to their expectations, open reduction and internal fixation is superior to percutaneous treatment in fracture union. A possible explanation for this result is that open reduction and internal fixation can remove foreign matter, such as a blood clot, crushed bone, and broken bone fragments in the tissue; therefore, more accurate reduction and sufficient pressure can better stimulate the increase of blood supply, thereby reducing the incidence of nonunion.^[10,20,21,50,59–62]^ Conversely, percutaneous fixation is performed blindly so a blood clot left in the tissue may result in inflammatory irritation to the blood supply of the scaphoid bone, resulting in less effective fracture union.^[50]^

### 4.3 Strengths and limitations

In addition to conventional outcome measures, this meta-analysis added the degree of patient satisfaction and pain that were ignored or insufficiently investigated in previous meta-analyses. Additionally, we included Chinese and English studies, RCTs, and cohort studies; expanded the number of primary documents; and increased data sources for outcomes. The GRADEpro system was also used to assess the quality of the evidence.

However, this meta-analysis still has the following limitations. First, different data structure standards reported by various literatures make it impossible to extract the data from some studies, resulting in an insufficient number of basic literatures. Second, because of the lack of age-related data in the included studies, we could not assess the effect of age on outcome measures for surgical and nonsurgical treatments among patients of different ages.^[63]^

## 5 Conclusions

For scaphoid waist fractures with slight or no displacement, there was no statistical difference in patient satisfaction, pain, and DASH scores between surgical treatment and nonsurgical treatment. Closed surgical treatment can shorten the time to union and convalescence, and open reduction can reduce the incidence of nonunion. On the basis of this conclusion, chief physicians can consider which treatment to use according to the patient's clinical situation and their subjective intention.

### 5.1 Implications for Future Research

Future research teams should focus on patient satisfaction and pain indicators, and further explore the effects of percutaneous and open surgery on the time to union, recovery, and incidence of nonunion to provide recommendations for treating scaphoid fractures. Furthermore, in future clinical studies, investigators should carefully design high-quality study protocols, pay attention to the different nature of the patients’ job type, and use standardized observation standards, measurement standards, and record forms in measurement analysis to develop a unified display method. Thus, the meta-analysis can lead to a precise conclusion.

## Author contributions

**Conceptualization:** Wenlai Guo, Shishun Zhao, Rui Li.

**Data curation:** Hangyu Li, Shishun Zhao.

**Investigation:** Hangyu Li, Wenlai Guo.

**Resources:** Shishun Zhao.

**Software:** Shanshan Guo.

**Writing – original draft:** Hangyu Li, Wenlai Guo, Shanshan Guo, Rui Li.

**Writing – review & editing:** Hangyu Li.

Shishun Zhao orcid: 0000-0002-1180-9708.

## Supplementary Material

Supplemental Digital Content

## References

[1] Schmidle G Ebner HL Klima G Time-dependent changes in bone healing capacity of scaphoid fractures and non-unions. J Anat 2018;232 suppl 3:908–18. 29488208 10.1111/joa.12795 PMC5979627

[2] Tielvan Buul MM Roolker W Broekhuizen AH The diagnostic management of suspected scaphoid fracture. Injury 1997;28:1. 9196618 10.1016/S0020-1383(96)00127-1

[3] Sauerbier M Müller M Scaphoid fractures: diagnosis, surgical approach, and complications. Zentralblatt Für Chirurgie 2007;132:W42. 17610183 10.1055/s-2007-981160

[4] Haddad FS Goddard NJ Acute percutaneous scaphoid fixation. A pilot study. J Bone Joint Surg Br 1998;80:95–9. 9460961 10.1302/0301-620x.80b1.8076

[5] Schaefer M Siebert HR Die kahnbeinfraktur. Der Unfallchirurg 2002;105:540–53. 12132194 10.1007/s00113-002-0446-z

[6] Gutow AP Percutaneous fixation of scaphoid fractures. J Am Acad Orthop Surg 2007;15:474. 17664367 10.5435/00124635-200708000-00004

[7] Bhat AK Acharya AM Manoh S A prospective study of acute undisplaced and minimally displaced scaphoid fractures managed by aggressive conservative approach. J Hand Surg Asian Pac Vol 2018;23:18. 29409429 10.1142/S2424835518500029

[8] Shen L Tang J Luo C Comparison of operative and non-operative treatment of acute undisplaced or minimally-displaced scaphoid fractures: a meta-analysis of randomized controlled trials. PLoS One 2015;10:e0125247. 25942316 10.1371/journal.pone.0125247 PMC4420279

[9] Rayan GM Fractures and nonunions of the scaphoid. J Oklahoma State Med Assoc 1996;89:315. 8885535

[10] Rettig AC Management of acute scaphoid fractures. Hand Clin 2000;16:381. 10955212

[11] Adams JE Steinmann SP Acute scaphoid fractures. Orthop Clin North Am 2007;38:229–35. vi. 17560405 10.1016/j.ocl.2007.02.004

[12] Dias JJ Brenkel IJ Finlay DB Patterns of union in fractures of the waist of the scaphoid. J Bone Joint Surg Br 1989;71:307–10. 2925752 10.1302/0301-620X.71B2.2925752

[13] Severo AL Cattani R Schmid FN Percutaneous treatment for waist and proximal pole scaphoid fractures. Rev Bras Ortop 2018;53:267–75. 29892575 10.1016/j.rboe.2018.03.004 PMC5993880

[14] Winston MJ Weiland AJ Scaphoid fractures in the athlete. Curr Rev Musculoskel Med 2017;10:38–44. 10.1007/s12178-017-9382-y PMC5344853 28251560

[15] Rettig ME Kozin SH Cooney WP Open reduction and internal fixation of acute displaced scaphoid waist fractures. J Hand Surg 2001;26:271–6. 10.1053/jhsu.2001.21524 11279573

[16] Bedi A Jebson PJ Havden RJ Internal fixation of acute, nondisplaced scaphoid waist fractures via a limited dorsal approach: an assessment of radiographic and functional outcomes. J Hand Surg Am 2007;32:326–33. 17336838 10.1016/j.jhsa.2007.01.002

[17] Yip HS Wu WC Chang RY Percutaneous cannulated screw fixation of acute scaphoid waist fracture. J Hand Surg Br 2002;27:42–6. 11895344 10.1054/jhsb.2001.0690

[18] Patillo DP Khazzam M Robertson MW Outcome of percutaneous screw fixation of scaphoid fractures. J Surg Orthop Adv 2010;19:114–20. 20727308

[19] Dehghani M Nekoei F Fatahi F Comparative study of results and complications of three methods in treatment of scaphoid fractures. J Isfahan Med School 2010;28:408–15.

[20] Ring D Jupiter JB Herndon JH Acute fractures of the scaphoid. J Am Acad Orthop Surg 2000;8:225–31. 10951111 10.5435/00124635-200007000-00003

[21] Buijze GA Doomberg JN Ham JS Surgical compared with conservative treatment for acute nondisplaced or minimally displaced scaphoid fractures: a systematic review and meta-analysis of randomized controlled trials. J Bone Joint Surg A 2010;92:1534–44. 10.2106/JBJS.I.01214 20516332

[22] Suh N Benson EC Faber KJ Treatment of acute scaphoid fractures: a systematic review and meta-analysis. Hand (N Y) 2010;5:345–53. 22131912 10.1007/s11552-010-9276-6 PMC2988115

[23] Symes TH Stothard J A systematic review of the treatment of acute fractures of the scaphoid. J Hand Surg Eur Vol 2011;36:802–10. 21700649 10.1177/1753193411412151

[24] Schädel-Höpfner M Marent-Huber M Sauerbier M Operative versus conservative treatment of non-displaced fractures of the scaphoid bone. Results of a controlled multicenter cohort study. Unfallchirurg 2010;113:806–13. 10.1007/s00113-010-1848-y 20827544

[25] Clementson M Jørgsholm P Besjakov J Union of scaphoid waist fractures assessed by CT scan. J Wrist Surg 2014;4:49–55. 10.1055/s-0034-1398472 PMC4327725 25709879

[26] Lin X Zeng J Guo Y Computer-assisted design of scaphoid reconstruction: individualized percutaneous cannulated screw fixation. Chin J Tissue Eng Res 2014;18:7178–82.

[27] Clementson M Jørgsholm P Besjakov J Conservative treatment versus arthroscopic-assisted screw fixation of scaphoid waist fractures - A randomized trial with minimum 4-year follow-up. J Hand Surg 2015;40:1341–8. 10.1016/j.jhsa.2015.03.007 25913660

[28] Moher D Liberati A Tetzlaff J Preferred reporting items for systematic reviews and meta-analyses: the PRISMA statement. PLoS Med 2009;6:e1000097. 19621072 10.1371/journal.pmed.1000097 PMC2707599

[29] Juni P Altman DG Egger M Systematic reviews in health care: assessing the quality of controlled clinical trials. BMJ 2001;323:42–6. 11440947 10.1136/bmj.323.7303.42 PMC1120670

[30] Jadad AR Moore RA Carroll D Assessing the quality of reports of randomized clinical trials: is blinding necessary? Control Clin Trials 1996;17:1–2. 8721797 10.1016/0197-2456(95)00134-4

[31] MacDermid JC Development of a scale for patient rating of wrist pain and disability. J Hand Ther 1996;9:178–83. 8784681 10.1016/s0894-1130(96)80076-7

[32] Atroshi I Gummesson C Andersson B The disabilities of the arm, shoulder and hand (DASH) outcome questionnaire: reliability and validity of the Swedish version evaluated in 176 patients. Acta Orthop Scand 2000;71:613–8. 11145390 10.1080/000164700317362262

[33] Rajagopalan BM Squire DS Samuels LO Results of Herbert-screw fixation with bone-grafting for the treatment of nonunion of the scaphoid. J Bone Joint Surg Am 1999;81:48–52. 9973053 10.2106/00004623-199901000-00007

[34] Xiaowen H Pu WK Xin C How to estimate the mean and standard deviation based on the median,range and sample size when conducting meta-analysis Chinese. Chin J Evid Based Med 2015;4:484–7.

[35] Higgins JPT Thompson SG Deeks JJ Measuring inconsistency in meta-analyses. Brit Med J 2003;327:557–60. 12958120 10.1136/bmj.327.7414.557 PMC192859

[36] Higgins JPT Thompson SG Quantifying heterogeneity in a meta-analysis. Stat Med 2002;21:1539. 12111919 10.1002/sim.1186

[37] Alnaeem H Aldekhayel S Kanevsky J A systematic review and meta-analysis examining the differences between nonsurgical management and percutaneous fixation of minimally and nondisplaced scaphoid fractures. J Hand Surg Am 2016;41:1135. 27707564 10.1016/j.jhsa.2016.08.023

[38] Rettig AC Weidenbener EJ Gloyeske R Alternative management of midthird scaphoid fractures in the athlete. Am J Sports Med 1994;22:711–4. 7810798 10.1177/036354659402200522

[39] Adolfsson L Lindau T Arner M Acutrak screw fixation versus cast immobilisation for undisplaced scaphoid waist fractures. J Hand Surg 2001;26 B:192–5. 10.1054/jhsb.2001.0558 11386765

[40] Bond CD Shin AY McBride MT Percutaneous screw fixation or cast immobilization for nondisplaced scaphoid fractures, Journal of bone and joint surgery. J Bone Joint Surg Am 2001;83-a:483–8. 11315775 10.2106/00004623-200104000-00001

[41] Saedén B Törnkvist H Ponzer S Fracture of the carpal scaphoid. A prospective, randomised 12-year follow-up comparing operative and conservative treatment. J Bone Joint Surg Br 2001;83:230–4. 11284571 10.1302/0301-620x.83b2.11197

[42] Dias JJ Wildin CJ Bhowal B Should acute scaphoid fractures be fixed? A randomized controlled trial. J Bone Joint Surg Am 2005;87:2160–8. 16203878 10.2106/JBJS.D.02305

[43] Arora R Gschwentner M Krappinger D Fixation of nondisplaced scaphoid fractures: making treatment cost effective. Prospective controlled trial. Arch Orthop Trauma Surg 2007;127:39–46. 17004075 10.1007/s00402-006-0229-z

[44] Vinnars B Ekenstam FA Gerdin B Comparison of direct and indirect costs of internal fixation and cast treatment in acute scaphoid fractures: a randomized trial involving 52 patients. Acta Orthop 2007;78:672–9. 17966028 10.1080/17453670710014383

[45] Vinnars B Pietreanu M Bodestedt A Nonoperative compared with operative treatment of acute scaphoid fractures. A randomized clinical trial. J Bone Joint Surg Am 2008;90/A:1176–85. 10.2106/JBJS.G.00673 18519309

[46] Dias JJ Dhukaram V Abhinav A Clinical and radiological outcome of cast immobilisation versus surgical treatment of acute scaphoid fractures at a mean follow-up of 93 months. J Bone Joint Surg Brit 2008;90:899–905. 18591600 10.1302/0301-620X.90B7.20371

[47] McQueen MM Gelbke MK Wakefield A Percutaneous screw fixation versus conservative treatment for fractures of the waist of the scaphoid: a prospective randomised study. J Bone Joint Surg Brit 2008;90:66–71. 18160502 10.1302/0301-620X.90B1.19767

[48] Brozek J Akl E Falck-Ytter Y 046 Guideline development tool (GDT)—web-based solution for guideline developers and authors of systematic reviews. BMJ Quality Safety 2013;22 suppl 1:82–182.

[49] Chen H Wang Y Hu XM How to use gradepro GDT to rate the quality of evidence in systematic reviews of intervention studies: An introduction. Chin J Evid Based Med 2015;15:600–6.

[50] Alshryda S Shah A Odak S Acute fractures of the scaphoid bone: systematic review and meta-analysis. Surgeon 2012;10:218–29. 22595773 10.1016/j.surge.2012.03.004

[51] O’Brien L Herbert T Internal fixation of acute scaphoid fractures: a new approach to treatment. Aust N Z J Surg 1985;55:387–9. 3870170 10.1111/j.1445-2197.1985.tb00906.x

[52] Skirven T Trope J Complications of immobilization. Hand Clinics 1994;10:53. 8188779

[53] Jincai L Long-term follow-up of 100 cases of scaphoid fracture. Chin J Hand Surg 1999;15:186–186.

[54] Brogan DM Moran SL Shin AY Outcomes of open reduction and internal fixation of acute proximal pole scaphoid fractures. Hand 2015;10:1–6. 26034435 10.1007/s11552-014-9689-8 PMC4447653

[55] Zlotolow DA Knutsen E Yao J Optimization of volar percutaneous screw fixation for scaphoid waist fractures using traction, positioning, imaging, and an angiocatheter guide. J Hand Surg Am 2011;36:916–21. 21527146 10.1016/j.jhsa.2011.02.017

[56] Garcia RM Ruch DS Management of scaphoid fractures in the athlete: open and percutaneous fixation. Sports Med Arthrosc Rev 2014;22:22–8. 24651287 10.1097/JSA.0000000000000008

[57] Wozasek GE Moser KD Percutaneous screw fixation for fractures of the scaphoid. J Bone Joint Surg Br 1991;73:138. 1670499 10.1302/0301-620X.73B1.1670499

[58] Slade J Jaskwhich D Percutaneous fixation of scaphoid fractures. Hand Clin 2001;17:553–74. 11775468

[59] Yang ZY Liu WJ Song ZJ Treatment of Herbert type I scaphoid fractures by percutaneous cancellous cannulted screw fixation Chinses. J Hand Surg 2005;21:154–5.

[60] Trumble TE Gilbert M Murray LW Displaced scaphoid fractures treated with open reduction and internal fixation with a cannulated screw. J Bone Joint Surg Am 2000;82:633–41. 10819274 10.2106/00004623-200005000-00004

[61] Segalman KA Graham TJ Scaphoid proximal pole fractures and nonunions. J Am Soc Surg Hand 2004;4:233–49.

[62] Ibrahim T Qureshi A Sulton AJ Surgical versus nonsurgical treatment of acute minimally displaced and undisplaced scaphoid waist fractures: pairwise and network meta-analyses of randomized controlled trials. J Hand Surg Am 2011;36:1759.e1–68.e1. 22036276 10.1016/j.jhsa.2011.08.033

[63] Alsawadi A Stanton J Scaphoid fracture in the elderly: a review. Hand Surg 2012;17:295–8. 22745103 10.1142/S0218810412300021

