# Supplementary figures and images for: Surgical versus nonsurgical treatment for scaphoid waist fracture with slight or no displacement: A meta-analysis and systematic review
Source: Medicine (Baltimore). 2018 Nov 30;97(48):e13266. doi: 10.1097/MD.0000000000013266 (PMC6283056; doi:10.1097/MD.0000000000013266)

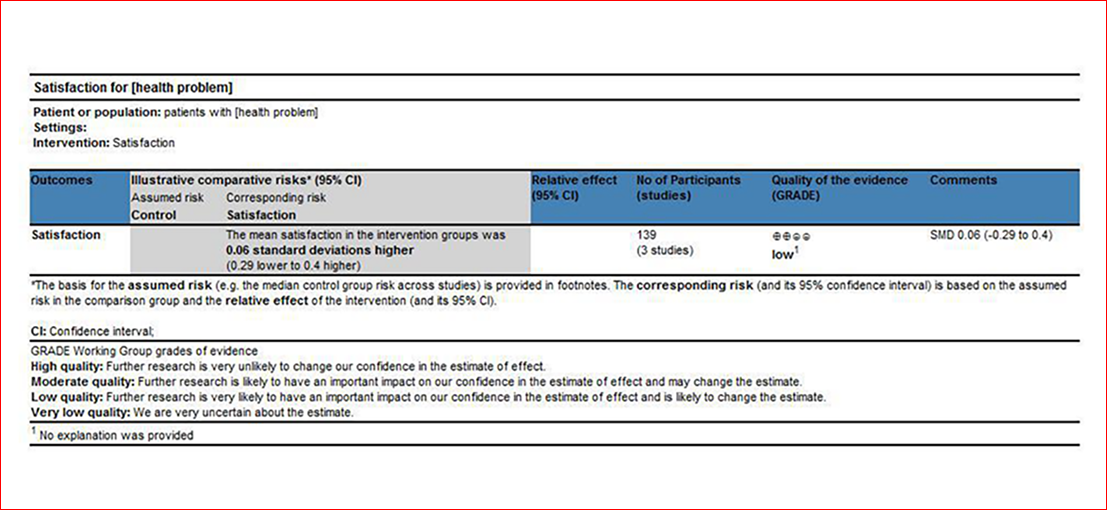


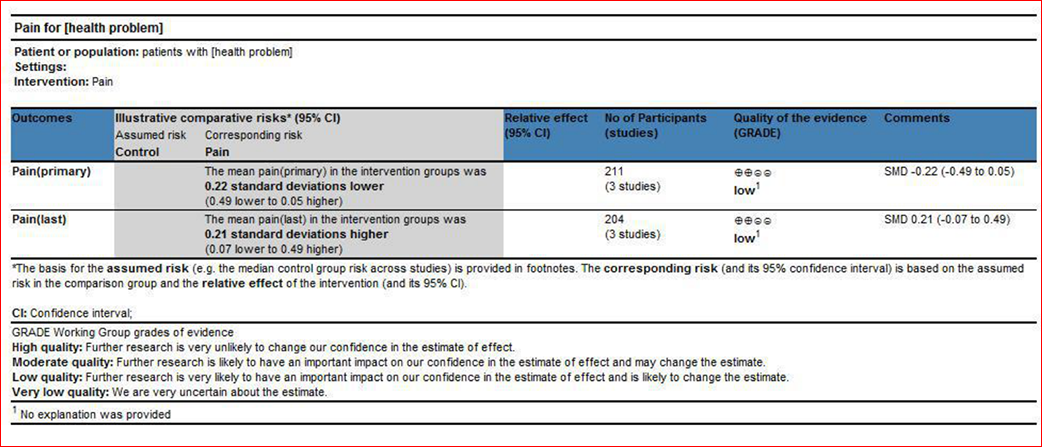


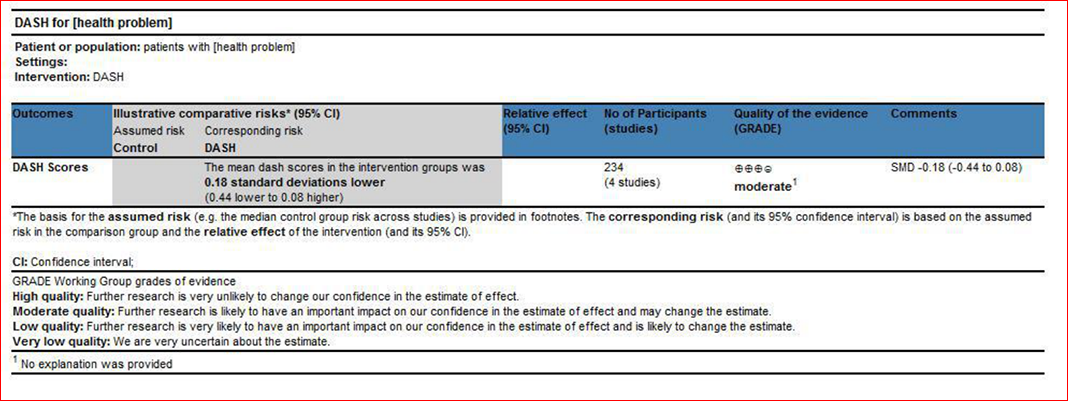


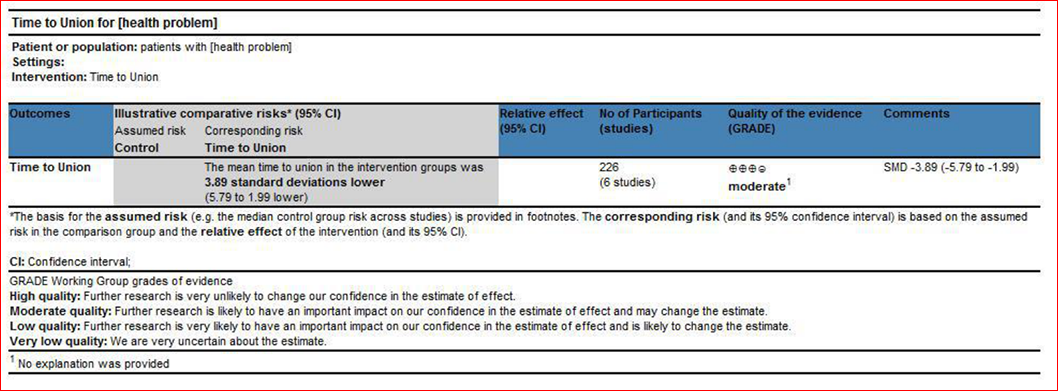


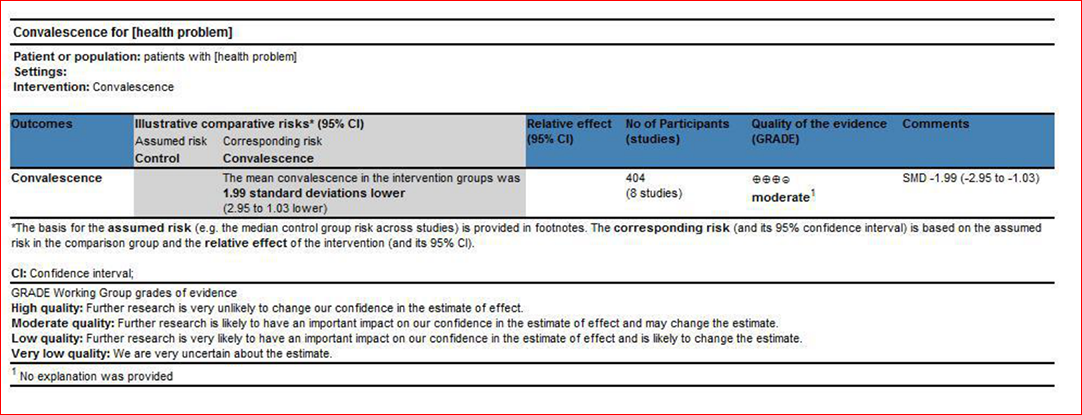


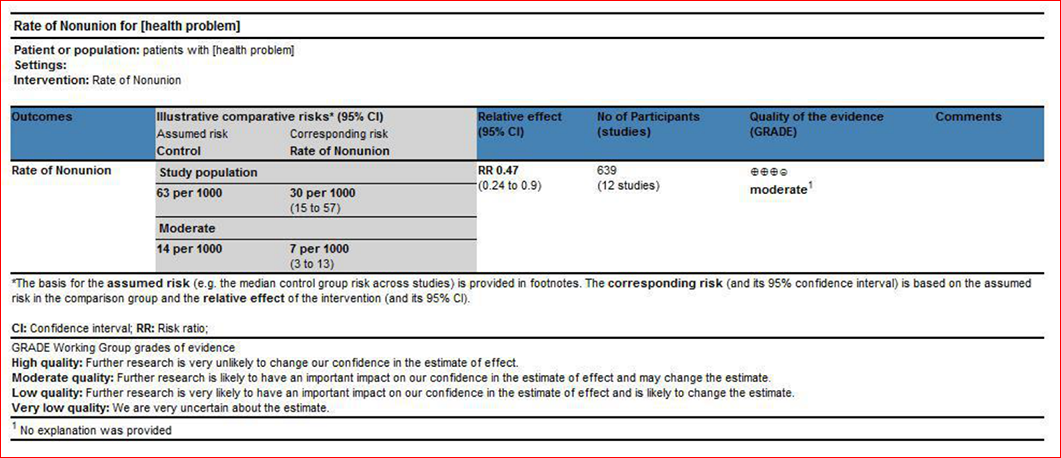

Supplement: Supplemental Digital Content [file medi-97-e13266-s001.doc]
